# An ideal dielectric coat to avoid prosthesis RF-artefacts in Magnetic Resonance Imaging

**DOI:** 10.1038/s41598-017-00215-7

**Published:** 2017-03-23

**Authors:** U. Zanovello, L. Matekovits, L. Zilberti

**Affiliations:** 1 Politecnico di Torino, I-10129 Torino, Italy; 2 Istituto Nazionale di Ricerca Metrologica, I-10135 Torino, Italy; 3 Macquarie University, NSW, 2109 Sydney Australia

## Abstract

The number of people submitted to total hip or knee arthroplasty increased in the last years and it is likely to grow further. Hence, the importance of a proper investigation tool that allows to determine and recognize the potential presence of perioperative and/or postoperative diseases becomes clear. Although the Magnetic Resonance Imaging (MRI) technique demonstrated several advantages over the other common tomography tools, it suffers from the arise of image artefacts if it is performed in presence of metallic prostheses. In particular, the so-called RF-artefacts are caused by the inhomogeneity in the radiofrequency magnetic field of MRI, due to the electric currents induced on the metal surface by the field itself. In this work, a near-zero permittivity dielectric coat is simulated to reduce those currents and, therefore, the RF-artefacts onset in the final image. Numerical results confirm that the dielectric coat strongly reduces the magnetic field inhomogeneity, suggesting a possible solution to a well-known problem in the MRI field.

## Introduction

The joint replacement procedure has become more and more frequent during the last decades. For example, the number of Americans living with a total hip or total knee replacement has increased from 540,000 (i.e. 0.11% of the U.S. population) in 1980, to over than 7,000,000 (i.e. a little over 2% of the U.S. population) in 2010. This number is likely to grow further in the next years and it has been estimated that the U.S. people submitted to a total hip or knee arthroplasty will reach 11,000,000 in 2030^1^.

Arthroplasty treatment procedures can introduce several orthopaedic complications. These complications may occur throughout the metallic implant life, not being limited to the perioperative period only. The wear between the primary bearing surfaces of a prosthesis may result in the release of prosthetic particles, inducing an immune response in the periprosthetic vicinity. Over time, this process could result in an excessive accumulation of bone-resorbing factor and finally in osteolysis. At the same time, also aseptic loosening is counted among the osteolysis causes and it has been recognized as a main reason for implants revisions^2^.

Magnetic Resonance Imaging (MRI) demonstrated to be an excellent tool for the evaluation of several pathologies resulting from a total hip arthroplasty. Indeed, although conventional radiography, computerized tomography (CT), nuclear scintigraphy and aspiration arthrography represent useful investigation tools, they suffer from poor soft tissue contrast, spatial resolution, sensitivity and/or specificity^3^. Furthermore, contrary to CT, MRI does not subject patients to any ionizing radiations dose. For these reasons, it is more and more involved in the assessment of the diseases due to a joint replacement procedure. In particular, MRI emerges as the most sensitive method to quantify the location and extent of osteolysis and as the optimal mean to image nerves surrounding hip arthroplasty^4^.

It has been demonstrated that non-ferromagnetic metallic objects are not affected by significant attraction or deflection forces during an MR procedure and therefore, from this viewpoint, they do not represent a safety issue^5^. However, non-ferromagnetic metallic objects may cause temperature increase and local artefacts in the final MR image. Whereas the first effect represents a safety problem, the second one may affect the correct interpretation of the exam results.

Briefly, the MRI operating principle is based on the application of a strong static magnetic field *B*_0_ (common strength values are from tenths of tesla to 3 T for scanners used for diagnostic purposes) to align the nuclear magnetization vectors in the investigated volume. If a proper radiofrequency (RF) magnetic field *B*_1_ is applied at Larmor frequency (which depends on the nucleus that is intended to be excited and scales linearly with *B*_0_) along a direction transverse to *B*_*0*_, the nuclear magnetization vectors start to rotate around the *B*_1_ direction. When the RF magnetic field is switched-off, they tend to re-align with the static field *B*_0_. During this process, the nuclear magnetization vectors precess around *B*_0_ with Larmor frequency, giving rise to a swarm of signals that can be recorded by means of suitable receiving coils. The characteristics of each elementary signal depend on the type of tissues where it is originated. Finally, by means of gradient field coils properly switched on and off, it is possible to introduce a spatial dependence in the signal features (and therefore to identify the position of the source of such signal), allowing the composition of the MR image.

Artefacts due to the presence of metallic objects affect the correct interpretation of the tomographic exam and may arise from several sources. The difference between the magnetic susceptibility of the metallic implants and the one of the periprosthetic tissues gives rise to *B*_0_ inhomogeneities that can result in geometric distortion, perturbations and signal intensity variations^6^. Susceptibility artefacts have been deeply studied in literature^7^ and several specific metal suppression techniques (e.g. “View Angle Tilting” (VAT), “Slice Encoding for Metal Artefact Correction” (SEMAC), “Multiple-acquisition with Variable Resonances Image Combination” (MAVRIC)) have been proposed to mitigate the unwanted effects^8^.

Another type of artefacts derives from the *B*_1_ inhomogeneities and seems to be quite less investigated in literature. In presence of a time dependent magnetic field, the eddy currents induced in a metallic object can generate a scattered magnetic field that overlaps the original *B*_1_. The resultant magnetic field depends on several parameters, such as the object shape, material, orientation and position relative to the RF coil. *B*_1_ inhomogeneities lead mainly to signal intensity variations^9, 10^.

Finally, another source for artefacts generation seems to be due to gradient switching^11, 12^. The physical principle that is responsible for the rising of such artefacts is the same as for RF-artefacts. In this case, the eddy currents circulating within non-zero conductivity objects are induced by gradient switching. These artefacts are strongly influenced, among others, by the applied MR sequence and the selected imaging parameters such as FOV and matrix size.

Currently, the most common alloys involved in the production of prostheses are cobalt-chromium (CoCr), titanium (Ti) and MR-compatible stainless steel. Cobalt-chromium is prevalently used for joint hard-ware characterized by important wear-inducing movements, titanium is often used for structural integrity and stainless steel is used for a combination of the two purposes^8^.

Titanium susceptibility value is five times lower than that of CoCr alloys. This means that, if RF-induced artefacts can be negligible compared to susceptibility artefacts in the case of a CoCr prosthesis, the situation may change in the imaging of a titanium implant. Furthermore, metal suppression techniques only address susceptibility artefacts, leaving RF-induced artefacts an unsolved problem. Taking into account, lastly, the trend towards stronger static magnetic fields^13^, an increment of the importance of the RF-induced artefacts has been registered with a *B*_0_ raise from 1.5 T to 3 T^9, 11, 14^ in clinical platforms, because of the corresponding increment in the RF field frequency.

Several solutions, more or less effective depending on the specific situation, have been proposed in order to mitigate the RF-induced artefacts^10^. Recently, Bachschmidt^15^ *et al*. proposed to change the *B*_1_ polarization depending on the position of the metallic implants inside the RF-coil. The proposed method seems to bring beneficial results, but suffers from the need to perform a preliminary *B*_1_ map on the patient in order to evaluate the optimal polarization.

The purpose of this work is to highlight the theoretical effects of the radiofrequency cloaking of metallic objects (mimicking a structural or joint prosthesis) from the RF electromagnetic field generated by a real MR antenna. In particular, the cloaking of a realistic hip prosthesis model is presented. The effective electric permittivity of a coat that minimizes the *B*_1_ inhomogeneities responsible for the rising of RF-induced artefacts is investigated. The benefit of such coat, compared to an uncloaked case, is demonstrated by means of numerical simulations. Finally, special care is given to the evaluation of the Specific Absorption Rate (SAR), defined as the power absorbed per unit of mass (see Methods section). Indeed, an excessive SAR value could cause tissue overheating with consequent damages and most of the limits imposed during an MRI procedure regard this quantity. It is known that the SAR distribution is influenced by several factors such as the human body size and shape, the frequency involved, the position of the prosthesis relative to the transmit coil, the prosthesis shape and dimensions and the prosthesis material. Often, a local enhancement of SAR is predicted near the hip prosthesis extremities and, in some situations, SAR values can even exceed the safety limits^16^. Taking into account those considerations, a specific analysis of SAR variation, when the coat is added to the metallic objects, is performed.

Possible artefacts and safety issues due to the gradient fields, are not considered in this work.

## Methods

**Figure 1:**
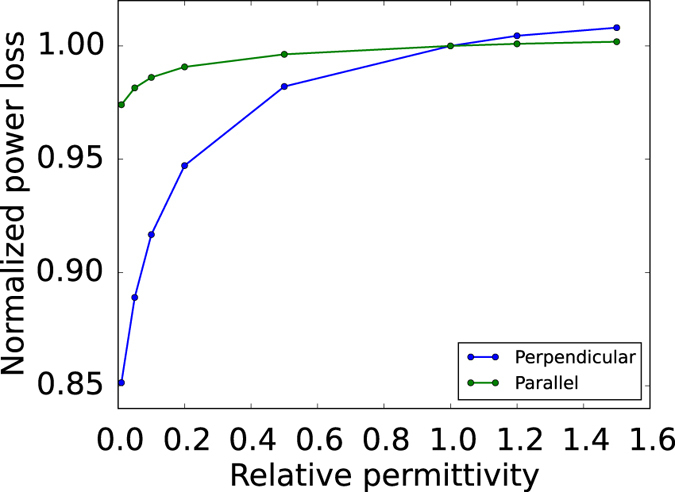
Preliminary analysis of the dielectric coat. Trend of the power loss on the titanium cylinder for different relative permittivity values of the dielectric coat. The power dissipated is normalized to the power loss without any coat. The “perpendicular” line refers to the case of a cylinder with its axis perpendicular to the direction of propagation of the electromagnetic wave; the “parallel” one to the case of a cylinder with its axis parallel to the direction of propagation of the electromagnetic wave.

The preliminary coat optimization was performed through a commercial simulation software (CST-MWS^©^). A titanium cylinder (electrical conductivity equal to 2.38·10^6^ S/m), was conceived to mimic a generic structural prosthesis. The cylinder, having a 40 mm diameter and a 300 mm height, was covered by an ideal, lossless, dielectric coat. The coat had a thickness of 1 mm, zero electrical conductivity and a relative magnetic permeability equal to one. The dielectric thickness was set once and for all, taking into account that the coat must not affect the prosthesis dimensions significantly. The resulting coated metallic object was radiated, in air, with an electromagnetic field at 128 MHz (the frequency associated to a 3 T static magnetic field for hydrogen excitation). The field was circularly polarized and it was generated by means of the “Plane Wave Source” provided within CST-MWS^©^. The source was set to generate a magnetic field of 10 µT, that is a reasonable value for an MRI RF signal. Finally, the choice to employ a circular polarized electromagnetic field is suggested by the physical process of precession. In fact, when the RF magnetic field *B*_1_ is applied to cause the precession, the nuclear magnetization vectors react only to the component of the *B*_1_ signal that rotates clockwise respect to the direction of *B*_0_ (*B*_1_^+^). Generally, to avoid useless power deposition inside the tissues, the MRI antennas are designed to produce a circularly polarized magnetic field. The optimization goal was to minimize the currents induced in the metallic object by the electromagnetic field. As underlined in the introduction, minimizing the induced currents is a way to avoid the field inhomogeneities that result in the RF-induced artefacts. In order to evaluate the whole currents circulating through the metallic cylinder, the active power dissipated inside the titanium was computed and used as optimization goal. Considering that the dissipated power is proportional to the square of the induced current intensity, the optimization goal could be equally addressed to the minimization of the titanium power loss. By means of the optimization tool offered by the software, an optimum relative electric permittivity of the coat was investigated in a range from 0.01 to 1.5, contemplating the employment of properly designed metamaterials in the case of relative permittivity lower than one. The optimization process was performed both with the main axis of the cylinder perpendicular to the direction of propagation of the electromagnetic field and with the axis parallel to the direction of propagation. In Fig. 1 the dissipated power, normalized to the power loss without any coat, is shown as a function of the relative permittivity.

The sensitivity of the dissipated power with respect to the variation of the coat permittivity is almost negligible when the cylinder axis is parallel to the direction of propagation (see green line of Fig. 1). Nevertheless, in both cases the power loss decrease is appreciable for low permittivity values. Whereas the dissipated power decreases with the electric permittivity reduction, the difficulty of achieving a metamaterial with those electric characteristics grows more and more. Thus, an electric permittivity equal to 0.1 was selected for the investigated coat. The reasons behind this choice, as well as the physical meaningfulness and feasibility of a near-zero permittivity material, are deepened in the Discussion section.

The coat was then used to cover three metallic objects with different shapes. The first exhibited a cylindrical geometry, mimicking an elongated structural prosthesis. The cylinder dimensions were the same used for the coat optimization phase. The second metallic object was a disc with a 40 mm radius and 20 mm height, rotated by 45° about one diameter. Its aim was to mimic the shape of implants like the acetabular cup of the hip prosthesis, or an intervertebral disc prosthesis.

The disc radial dimension was intentionally overestimated, to stress the edge effects and magnetic field perturbation.

Finally, the model of a realistic femoral stem (220 mm height) of a hip prosthesis was considered.

These three objects were simulated as perfect electric conductors (PEC). This choice decreases the simulation time without bringing to significant errors. In fact, at radiofrequencies, the electromagnetic behaviour of metals approaches that of PEC, making the proposed approximation reliable. It should be specified that the use of PEC inevitably affected the estimation of the power dissipated on the metal surface. However, hereafter, the coat effects were investigated in terms of *B*_1_ homogeneity and SAR distribution and the information about the power loss became useless.

**Figure 2:**
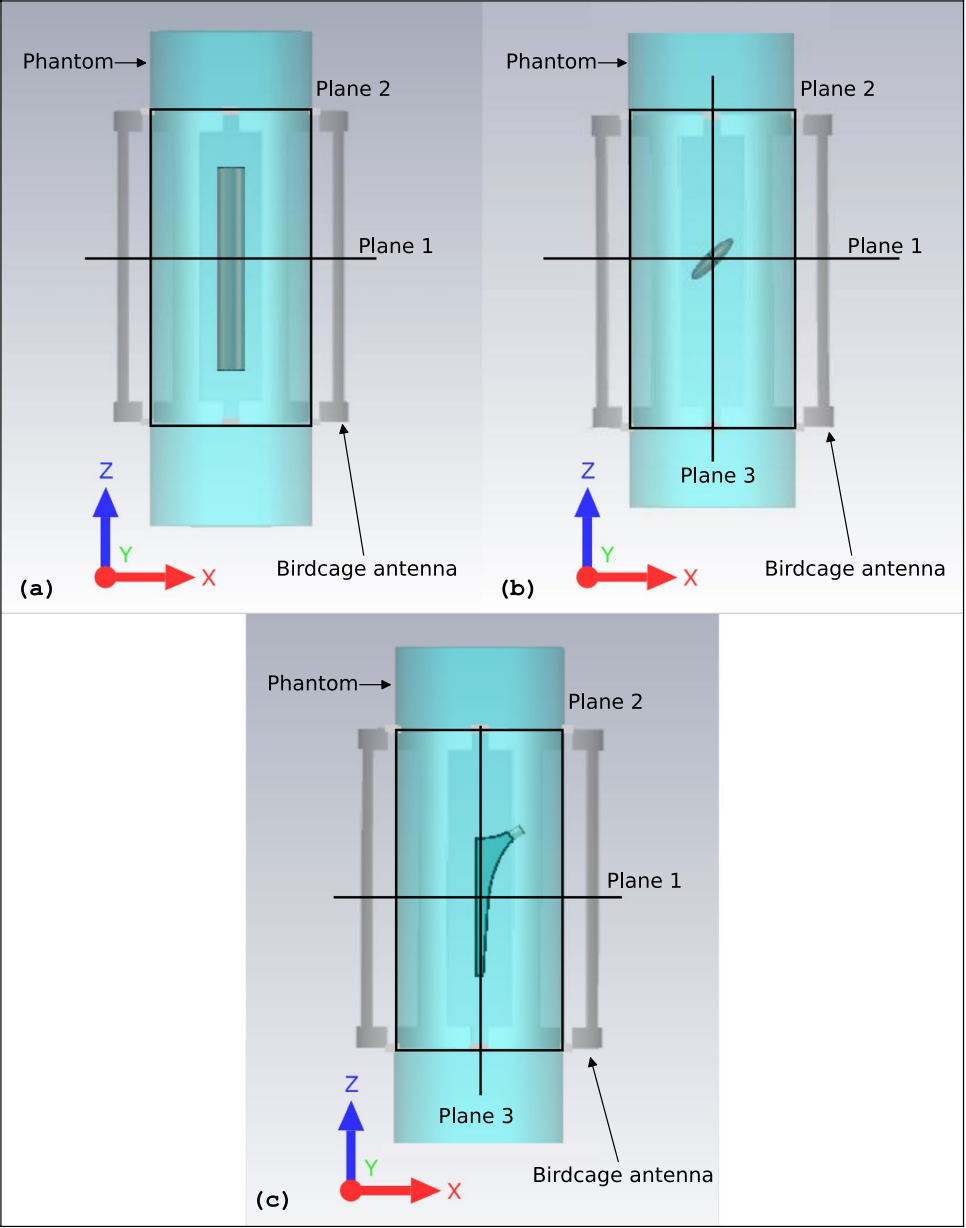
Simulation set-ups. Simulation set-ups relative to the PEC cylinder (**a**), PEC disc (**b**) and to the realistic model of a hip prosthesis (**c**) placed in the centre of the phantom transverse plane. The planes on which the magnetic field homogeneity and SAR were investigated are depicted. Considering the hip prosthesis model (**c**), plane 2 corresponds to the coronal plane and plane 3 to the sagittal one.

In the simulation environment, the metallic objects were alternately plunged into a cylindrical phantom (120 mm radius, 730 mm height) with a relative electric permittivity of 61.5 and an electrical conductivity of 0.87 S/m. Such values are close to those characterizing some of the human tissues^17^ at the frequency of interest and correspond to the electrical properties of a tissue-simulating liquid available in our laboratory. The cylindrical phantom was radiated by a volume coil similar to those employed in a real MR scanner^18^. The antenna (i.e. the coil) was an 8-legs birdcage with a 175 mm radius and 460 mm height and it was driven in quadrature mode (in order to generate a circularly polarized magnetic field) at 128 MHz (Fig. 2). Open boundary conditions were applied all around the computational domain.

In order to obtain the unperturbed situation, a preliminary simulation was performed without any metallic object inside the phantom. The power outgoing from each port was set to obtain, in the unperturbed situation, a clockwise rotating magnetic field of 2.5 µT in the centre of the coil. This power was used to feed the coil in every examined set-up.

After that, the metallic objects were separately plunged into the phantom. They were simulated in the longitudinal centre of the phantom and for two different positions in the transverse plane. In the first case, the objects were placed in the centre of the transverse plane, whereas in the second case they were shifted by 80 mm on the same transverse plane towards the phantom lateral surface.

All simulations were performed with and without the coat to investigate the consequences of its presence. All the effects were assessed both in terms of the clockwise rotating magnetic field (*B*_1_^+^) homogeneity inside the phantom and in terms of SAR variation with respect to the case without prosthesis. Both quantities were evaluated on all the planes reported in Fig. 2. The simulation set-ups described above and not depicted in this paper, are available in the Supplementary material (Figs S1 and S2).

The clockwise rotating component of the magnetic field was computed as:

1 $${B}_{1}^{+}(P)=|\frac{\overline{{B}_{x}(P)}+j\overline{{B}_{y}(P)}}{2}|$$

where $\overline{{B}_{x}(P)}$ and $\overline{{B}_{y}(P)}$ are the phasors of the x-component and y-component (with the z-axis of the Cartesian coordinate reference aligned along the longitudinal axis of the phantom) of the magnetic field in point *P* and *j* is the imaginary unit.

Given the mass density (*ρ*(*P*)) and the electrical conductivity (*σ*(*P*)) of a tissue in a point *P*, the SAR can be evaluated as:

2 $$SAR=\frac{{\sigma }(P){\overline{E(P)}}^{2}}{{\rho }(P)}$$

where $\overline{E(P)}$ is the rms value of the electric field in point *P*. The safety limits generally distinguish between the whole body SAR (averaged over the mass of the whole body) and the local SAR (averaged over a mass of 10 g)^19, 20^. The local SAR average process reduces the incidence of the numerical errors when evaluating confined SAR hotspots. This is the reason why safety limits are not imposed on the SAR directly obtained by equation (2). In this work the SAR was computed with equation (2) (avoiding the uncertainty brought by the chosen averaged mass).

All the simulations were performed by means of the frequency-domain solver of CST-MWS^©^. In order to investigate the reliability of the obtained results, some of them were compared to those obtained through another commercial software, Sim4Life^©^, based on a finite-difference time-domain solver. The comparisons were performed both for the SAR distribution and for the clockwise rotating magnetic field generated inside the coil with the uncoated cylindrical object plunged into the centre of the phantom. The agreement between the results was found to be satisfactory for both quantities. In the Supplementary material (Figs S3 and S4) such comparisons are presented along a diametrical line crossing the transverse plane (“Plane 1” of Fig. 2(a)).

In the result section, the homogeneity of *B*_1_^+^ on a given slice, normalized with respect to the maximum *B*_1_^+^ of the same slice, is proposed:

3 $${{B}_{1}^{+}(P)|}_{d{B}_{MAX}}=20\,{\mathrm{log}}_{10}\frac{{B}_{1}^{+}(P)}{{{\rm{\max }}({B}_{1}^{+})|}_{{Slice}}}$$

where ${B}_{1}^{+}(P)$ represents the clockwise component of the magnetic field in point *P* (see equation (1)) and ${{\rm{\max }}({B}_{1}^{+})|}_{{Slice}}$ is the maximum of ${B}_{1}^{+}(P)$ on the considered slice.

In order to compare the homogeneity of *B*_1_^+^ on the given slice between different set-ups, the standard deviation of this quantity was computed and reported. The standard deviation was evaluated both considering the whole slice and only a subregion (whose area and shape depend on the specific set-up) surrounding the object. The zones outside the slice and inside the metallic objects (when present) were excluded from the computation of the standard deviation.

In order to highlight the possible critical SAR issues brought by the coated objects, the SAR values were compared to those obtained for the unperturbed case (i.e. with the empty phantom).

In the result section, the SAR ratio is evaluated with the following expression:

4 $${{SAR}(P)|}_{dB}=10\,{\mathrm{log}}_{10}\frac{{{SAR}(P)|}_{2{WAvg}}^{{WithObject}}}{{{SAR}(P)|}_{2{WAvg}}^{{WithoutObject}}}$$

where ${{SAR}(P)|}_{2{WAvg}}^{{WithObject}}$ and ${{SAR}(P)|}_{2{WAvg}}^{{WithoutObject}}$ represent the local SAR value in point *P* with and without the metallic object, respectively. In both cases, the data were preliminarily rescaled to get an average SAR of 2 W/kg over the whole phantom. 2 W/kg represents the limit established by the IEC 60601-2-33 standard^20^ for the whole-body SAR in normal operating mode.

## Results

**Figure 3:**
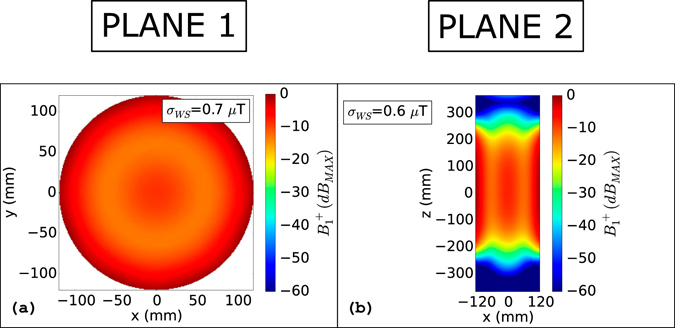
*B*_*1*_^+^(*dB*_*MAX*_): Unperturbed condition. *B*_*1*_^+^(*dB*_*MAX*_) evaluated for the ideal case without any object inside the coil. The figure refers to the transverse symmetry plane of the phantom (**a**) and to the longitudinal one (**b**). σ_WS_ represents the standard deviation computed on the whole slice.

**Figure 4:**
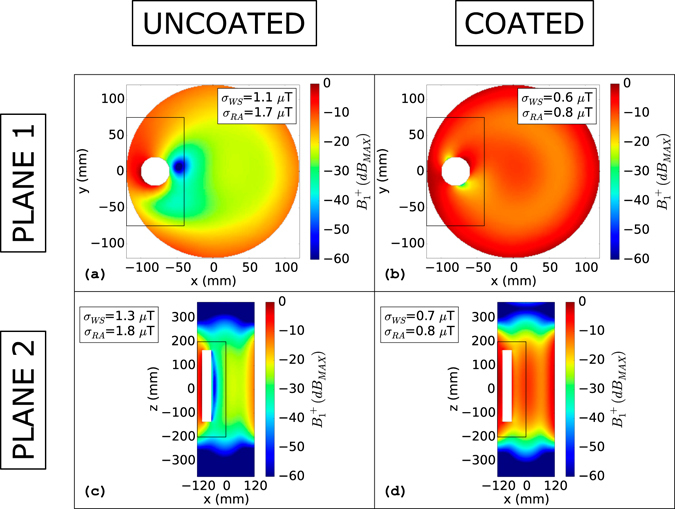
*B*_*1*_^+^(*dB*_*MAX*_): Lateral metallic cylinder. *B*_*1*_^+^(*dB*_*MAX*_) evaluated for the cylinder plunged into the phantom in a lateral position (−80 mm from the phantom centre to the cylinder centre). Figures (**a**) and (**b**) depict the uncoated and coated object situations, respectively, for the transverse plane that cuts the cylinder in two equal parts along the longitudinal direction. Figures (**c**) and (**d**) depict the uncoated and coated object conditions, respectively, for the longitudinal plane that cuts the phantom in two equal parts. The rectangular areas represent the reduced areas in which the standard deviation (σ_RA_) was computed. σ_WS_ represents the standard deviation computed on the whole slice.

The *B*_1_^+^ maps for the ideal case without any object inside the coil are depicted in Fig. 3. The map corresponding to “Plane 1” is reported in Fig. 3(a). Since, for the unperturbed set-up, there are no appreciable differences between “Plane 2” and “Plane 3” of Fig. 2(b),(c), only “Plane 2” is reported in Fig. 3(b). The standard deviation of the magnetic field on the transverse slice is equal to 0.7 µT. Considering the longitudinal plane of Fig. 3(b), the standard deviation of the magnetic field is equal to 0.6 µT on the whole slice. It should be specified that, dealing with the longitudinal planes, the whole slice standard deviation computation has been limited in the z direction from z = −200 mm to z = 200 mm. This choice removes from the analysis the *B*_1_^+^ inhomogeneities due to the finite length of the coil. These values do not change significantly, both for “Plane 1” and “Plane 2” of Fig. 2(a), in presence of the metallic cylinder plunged into the phantom centre (see Supplementary material: Fig. S5). The independence of the *B*_1_^+^ standard deviation from the presence of the metallic cylinder suggests that no sensible artefacts would be recorded during the scan of an elongated prosthesis symmetrically positioned in the centre of the coil supplied in quadrature operation. This consideration is consistent with the results obtained by Bachschmidt^15^ *et al*. The situation is analogous when the cylinder is replaced by the metallic disc or by the hip prosthesis model. Also in these cases, no sensible *B*_1_^+^ perturbation, in terms of standard deviation computed over the whole slice, is detected in the presence of the object (see Supplementary material: Figs S6 and S7). The *B*_1_^+^ maps for the metallic cylinder moved in a side position with respect to the phantom, are depicted in Fig. 4. As expected, the asymmetry due to the object strongly compromises the *B*_1_^+^ homogeneity both for “Plane 1” (Fig. 4(a)) and “Plane 2” (Fig. 4(c)). The standard deviations of the clockwise rotating magnetic field, evaluated on the whole slice, becomes 1.1 µT for “Plane 1” and 1.3 µT for “Plane 2”. The increase of the standard deviation is even more highlighted in the vicinity of the cylinder. Considering the rectangular areas depicted in Fig. 4 (x: −120 mm to −40 mm, y: −75 mm to 75 mm for “Plane 1” and x: −120 mm to 0 mm, y: −200 mm to 200 mm for “Plane 2”) the standard deviation grows to 1.7 µT for “Plane 1” and to 1.8 µT “Plane 2”. These values correspond to an increase of 150% (for “Plane 1”) and 170% (for “Plane 2”) with respect to the standard deviations evaluated on the same zones for the ideal case without any object inside the phantom. The effects of coating the cylinder with a proper dielectric coat are appreciable in Fig. 4(b),(d). The *B*_1_^+^ homogeneity becomes comparable to the one obtained for the unperturbed case (see Fig. 3). The standard deviations decrease to 0.6 µT for “Plane 1” and to 0.7 µT for “Plane 2” being comparable to those obtained for the ideal case without metallic objects. Considering the same rectangular area used to compute the standard deviations near the cylinder for the uncoated cases, they decrease by 55% both for “Plane 1” and for “Plane 2”.

**Figure 5:**
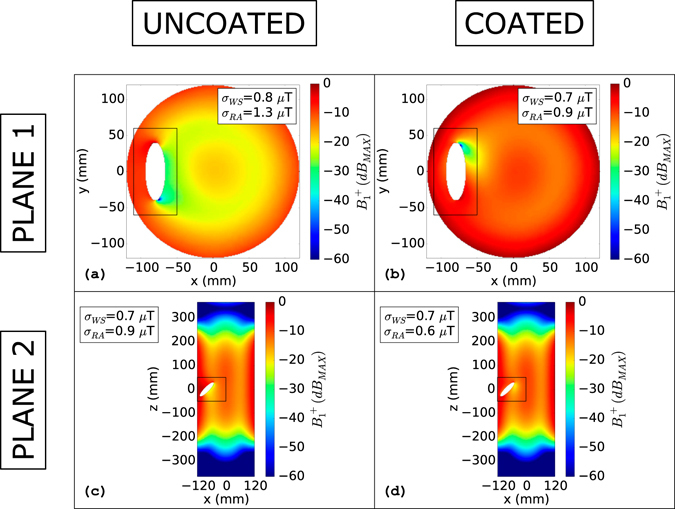
*B*_*1*_^+^(*dB*_*MAX*_): Lateral metallic disc. *B*_*1*_^+^(*dB*_*MAX*_) evaluated for the disc plunged into the phantom in a lateral position (−80 mm from the phantom centre to the disc centre). Figures (**a**) and (**b**) depict the uncoated and coated object situation, respectively, for the transverse plane that cuts the disc in two equal parts along the longitudinal direction. Figures (**c**) and (**d**) depict the uncoated and coated object condition, respectively, for the longitudinal plane that cuts the phantom in two equal parts. The rectangular areas represent the reduced areas in which the standard deviation (σ_RA_) was computed. σ_WS_ represents the standard deviation computed on the whole slice.

The situation is similar but less evident considering the lateral metallic disc. Taking into account the transverse symmetry plane (“Plane 1” of Fig. 2(b)) depicted in Fig. 5(a),(b), the standard deviation computed on the whole slice is 0.8 µT for the uncoated disc and it is reduced by 12% adding the coat around the disc. Considering a rectangular area surrounding the disc on the same plane (x: −110 mm to −50 mm, y: −60 mm to 60 mm) the positive effect becomes more appreciable. The standard deviation decreases by 31% passing from 1.3 µT to 0.9 µT when the coat is added. The *B*_1_^+^ inhomogeneities due to the metallic disc are more muffled considering the “Plane 2” in Fig. 5(c),(d). In this case the whole-slice standard deviation is 0.7 µT for the uncoated disc (Fig. 5(c)) and does not sensibly change for the coated disc (Fig. 5(d)). Also in this case, the coat effects are more appreciable considering a rectangular area surrounding the disc in “Plane 2” (x: −120 mm to 0 mm, z: −50 mm to 50 mm). In particular, the standard deviation decreases by 33% passing from 0.9 µT to 0.6 µT adding the coat. The results relative to “Plane 3” for the disc are shown and commented in the Supplementary material (Fig. S8).

**Figure 6:**
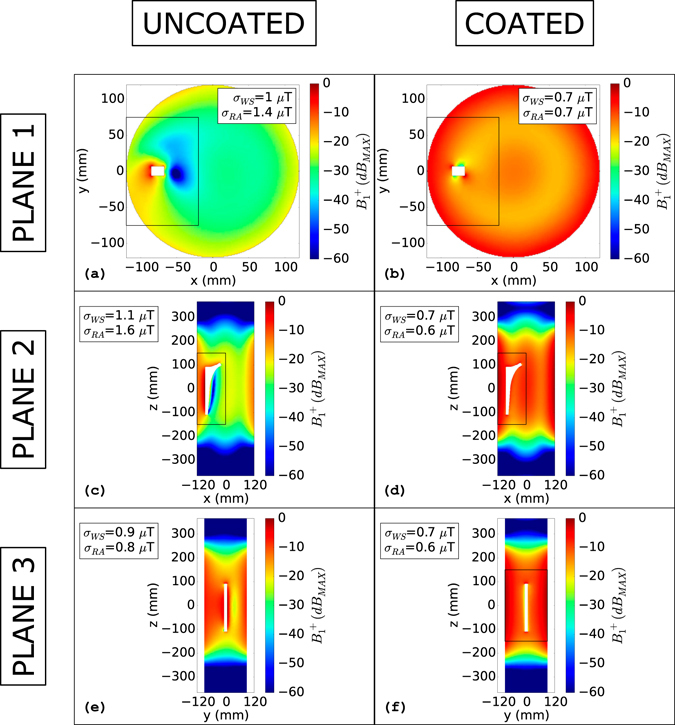
*B*_*1*_^+^(*dB*_*MAX*_): Lateral metallic hip prosthesis. *B*_*1*_^+^(*dB*_*MAX*_) evaluated for the realistic hip prosthesis model plunged into the phantom in a lateral position (−80 mm from the phantom centre to the prosthesis base centre). Figures (**a**) and (**b**) depict the uncoated and coated object situation, respectively, for the transverse plane that cuts the phantom in two equal parts along the longitudinal direction. Figures (**c**) and (**d**) depict the uncoated and coated object condition, respectively, for the coronal plane that cuts the phantom in two equal parts. Figures (**e**) and (**f**) depict the uncoated and coated object condition, respectively, for the sagittal plane that passes through the prosthesis base centre. The rectangular areas represent the reduced areas in which the standard deviation (σ_RA_) was computed. σ_WS_ represents the standard deviation computed on the whole slice.

In Fig. 6, the effects of the coat on the realistic prosthesis model are highlighted for all three planes of Fig. 2(c). Considering “Plane 1” of Fig. 2(a), the coat effects are noticeable. The standard deviation computed on the whole slice decreases from 1 µT (Fig. 6(a)) to 0.7 µT (Fig. 6(b)) adding the coat. The same parameter, computed in a reduced rectangular area (x: −120 mm to −20 mm, y: −75 mm to 75 mm), decreases by 50% passing from 1.4 µT to 0.7 µT. The coat effects remain evident considering a coronal plane (“Plane 2” of Fig. 2(c)). Considering the whole slice, the standard deviation decreases by 36%, passing from 1.1 µT (Fig. 6(c)) to 0.7 µT (Fig. 6(d)) when the coat is added. The decrease is even stronger if a reduced rectangular area, embracing the prosthesis section, is considered. The standard deviation reduces from 1.6 µT to 0.6 µT decreasing by more than 60%. Also in this case, the coat has the effect of making the object “invisible” to the surrounding field. In fact, when the coat is added, the standard deviation values become comparable to those obtained for the unperturbed case and, mainly, the “original” field distribution is almost restored. Figure 6(e),(f) show the coat effects on a coronal plane (“Plane 3” of Fig. 2(c)). In this plane, the coat effects are less appreciable. This is probably due to the small sagittal dimension of the prosthesis compared to those of the phantom. The standard deviation decreases from 0.9 µT to 0.7 µT considering the whole slice. Taking into account a smaller rectangular area (x: −90 mm to 90 mm, z: −150 mm to 150 mm), the standard deviation is reduced from 0.8 µT to 0.6 µT.

**Figure 7:**
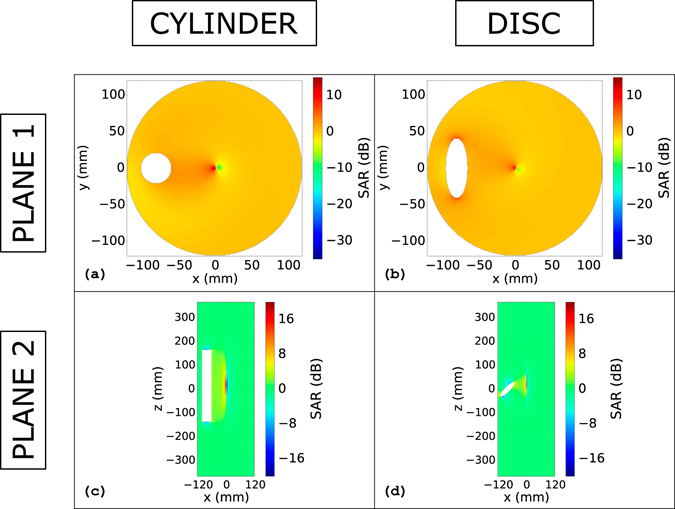
SAR: Lateral coated cylinder & lateral coated disc. Ratio between the SAR distribution with the coated metallic cylinder (**a**),(**c**) and disc (**b**),(**d**) and the one without any object. The figures refer to the objects positioned in a lateral side of the phantom (−80 mm from the phantom centre). Figures (**a**) and (**b**) refer to the transverse plane that cuts the object in two equal parts along the longitudinal direction. Figures (**c**) and (**d**) refer to the longitudinal plane that cuts the phantom in two equal parts.

**Figure 8:**
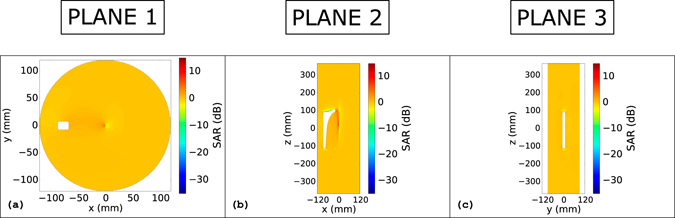
SAR: Lateral coated hip prosthesis. Ratio between the SAR distribution with the coated metallic hip prosthesis inside the phantom and the one without any object. Figures refer to the object plunged into a lateral position inside the phantom. Figure (**a**) refer to the transverse plane that cuts the phantom in two equal parts along the longitudinal direction. Figures (**b**) and (**d**) refer to the coronal and sagittal plane, respectively, that cut the prosthesis in two equal parts.

Finally, the ratio expressed in decibel, between the SAR with the coated metallic objects and without them are depicted in Fig. 7 and in Fig. 8 on the same planes investigated for the *B*_1_^+^ homogeneity. As explained in the Methods section, the SAR was preliminarily rescaled to get an average SAR of 2 W/kg over the whole phantom.

Similarly to what happens for the *B*_1_^+^ homogeneity, the SAR distribution does not seem to be heavily influenced by the presence of any of the considered objects when they are positioned in the phantom centre.

The SAR distribution for the “Plane 1” of Fig. 2(a),(b) is depicted in Fig. 7(a) for the cylinder in a lateral position and in Fig. 7(b) for the disc. The SAR distribution for “Plane 2” is shown in Fig. 7(c) for the metallic cylinder. Figure 7(d) represents the SAR distribution on “Plane 2” for the metallic disc. In Fig. 7(a) it is appreciable an increment of the SAR value with respect to the ideal case at the centre of the phantom. However, taking into account the low SAR values in the phantom centre for the ideal case, this increment should not bring to critical values from a safety point of view, as appreciable in Fig. S9 in the Supplementary material. The same considerations hold for the SAR distribution on “Plane 2” (Fig. 7(c)) for the cylinder and for the disc (Fig. 7(d)). In Fig. 7(b), an increment of the SAR values, with respect to the unperturbed case, is appreciable near the disk edges. This increment is found to be higher than that caused by an uncoated prosthesis.

Finally, the same SAR ratio distribution is depicted in Fig. 8 for the realistic hip prosthesis model positioned laterally in the phantom. The coat seems to not bring sensible SAR changes with respect to the unperturbed case. In Fig. 8(b), a SAR increment is appreciable in the centre of the slice. Again, the SAR ratio is enhanced by the low values that the SAR reaches in this zone and the increment remains lower than that introduced by the presence of the uncoated hip prosthesis (see Supplementary material: Fig. S10). The SAR distribution of the centred hip prosthesis is proposed in Fig. S11 of the Supplementary material.

## Discussion

The results highlight the benefits that a properly tailored coat would have on removing the RF magnetic field inhomogeneities due to the presence of a metallic object inside a phantom irradiated by an MRI volume coil antenna. The *B*_1_^+^ inhomogeneity strongly depends on the object shape and position relative to the coil. In particular, the more the metallic object is displaced from the phantom centre, the more the magnetic field inhomogeneity is enhanced. Furthermore, sharper objects tend to lead to magnetic field hot spots near their edges. A near-zero permittivity dielectric coat seems to reduce the currents induced by the RF electromagnetic field inside the metallic objects. These currents are responsible for the generation of a scattered magnetic field that compromises the original field homogeneity, resulting in RF-artefacts in the final tomographic image. In every examined case, the dielectric coat had the effect of reducing the *B*_1_^+^ standard deviation both on the investigated plane and on a smaller area surrounding the object. Covering the metallic objects with the coat results in a standard deviation reduction of more than 50% in the most critical situations, bringing it to be comparable to the same quantity evaluated with the ideal set-up (i.e. without any metallic object inside the phantom).

Whereas the induced currents reduction always has beneficial effects in terms of magnetic field homogeneity, the situation is more critical dealing with the SAR evaluation, for which the benefits of the coat remain ambiguous. Indeed, although it seems that the coat does not introduce “dangerous” SAR points, it leads to SAR increments, with respect to the ideal case, in some areas and, in some places, even with respect to the uncoated object. This happens because the electric currents induced on the metal surface could reduce the electric field at the object boundaries. Thus, minimizing those currents could not be the proper way to minimize the SAR around the metallic entity. It should be specified that the zones affected by SAR elevation are quite small in terms of area and the average process, on which the SAR threshold limits are based, could mitigate the SAR increment. Finally, it is worth mentioning that, differently from literature^16^, no appreciable local SAR enhancements were found considering the examined objects, neither for the uncoated objects cases. This is probably attributable to the different simulation set-up and it remains an aspect to be further investigated.

The crucial point of the proposed dielectric coat concerns its practical realization. The proposed results were obtained considering a dielectric coat with relative permittivity equal to 0.1. This value was chosen to present the beneficial effects that a prosthesis cloaking may have in reducing RF-induced artefacts. At this level, it is not possible to establish if the chosen relative permittivity value represents an optimum for the cloaking application and neither if such optimum univocally exists.

Apart from applications at infrared and optical frequencies, an epsilon-near-zero (ENZ) material needs in principle to be synthetized as metamaterial. The application of metamaterials in MRI environments is a topic that has been more and more investigated in the last years^21–23^ and particular applications have been recently proposed to be applied to this topic^24^. Furthermore, a great interest for electromagnetic field cloaking applications has been demonstrated for a wide range of frequencies. Magnetic and electric field invisibility cloaks have been proposed and demonstrated for static and low frequency applications^25–28^. On the other hand, several efforts have been done to apply metamaterials for electromagnetic cloaking at microwave frequencies^29, 30^.

The fundamental importance of ENZ media for cloaking applications has been recognized^31^ and their realization by means of metamaterials, properly tailored to obtain the desired electromagnetic behaviour in a chosen frequency bandwidth, has been a topic of great interest in the last years^32, 33^. Unlike the referenced cases, the application proposed in this paper presents several design constraints that should be taken into account. Firstly, the coat must not lead to a significant variation of the geometry and dimension of the prosthesis. It means that the coat thickness must be limited in the external direction evolving inwards at most. In every case, obviously, the coated prosthesis should present mechanical characteristics (e.g. yield strength, tensile strength, elastic modulus etc.) comparable to those of the uncoated one. Furthermore, the materials applied for the coat realization should be totally biocompatible and not subjected to degradation during the prosthesis life. Finally, some considerations about the coat losses are worth to be mentioned. In the proposed simulation, the coat was modelled as a lossless dielectric. Although some example of all-dielectric metamaterials is found in literature^34, 35^, the design principle of metamaterials is generally based on the periodical repetition of subwavelength unit cells composed by a conductive strip soldered on a dielectric substrate. The electric currents induced on the conductive path of the metamaterial could cause variations of the results obtained for the ideal coat in the proximity of the cloaked object.

The narrowband nature of metamaterials (i.e. the frequencies at which they show the desired electromagnetic behaviour) perfectly fits an MRI application where the frequency of the *B*_1_ field is univocally determined and limited in a range of a few tens of kilohertz. However, cloaking applications designed for the frequency range characterizing the RF magnetic field of MRI seem to lack in literature. Although the realization of a material whose characteristics approach those of the proposed coat represents an open issue at the moment, the aim of this work remains to demonstrate the benefits that a possible cloaking application would have on a civil and common procedure like MRI.

## Electronic supplementary material

Supplementary material
