# Supplementary material for: An ideal dielectric coat to avoid prosthesis RF-artefacts in Magnetic Resonance Imaging
Source: Sci Rep. 2017 Mar 23;7:326. doi: 10.1038/s41598-017-00215-7 (PMC5428001; doi:10.1038/s41598-017-00215-7)
Supplement: Supplementary file 1 — Supplementary material [file 41598_2017_215_MOESM1_ESM.pdf]

AN IDEAL DIELECTRIC COAT TO AVOID PROSTHESIS RF-ARTEFACTS IN MAGNETIC RESONANCE IMAGING

Umberto Zanovello[1,2], Ladislau Matekovits[1,3], and Luca Zilberti[2]

[1]Politecnico di Torino (I-10129 Torino, Italy)
[2]Istituto Nazionale di Ricerca Metrologica (I-10135 Torino, Italy)
[3]Macquarie University (NSW-2109 Sydney, Australia)

Simulation set-ups

In this section, the simulation set-ups and perspective not shown in the main paper are presented. Figure S1 shows the front view of the simulation set-ups with the metallic objects positioned in a lateral position (80 mm from the phantom centre). Figure S2 shows the top views of the simulation set-ups both for the objects in a central and lateral position.

Comparison between numerical codes

In this section, the comparison between the results obtained by means of two different numerical codes (i.e. Sim4Life© and CST-MWS©) is presented. Sym4Life© is a simulation platform, developed by ZurichMedTech, based on an finite-difference time-domain (FDTD) solver. In contrast, the results obtained with CST-MWS© are based on a frequency-domain simulation. The comparison was performed both in terms of the clockwise rotating magnetic field (B_1^+) and Specific Absorption Rate (SAR) along a phantom symmetry transverse line lying on “Plane 1” of Fig. S1. The considered simulation set-up consists of the uncoated cylindrical metallic object positioned in the centre of the phantom and irradiated by the Birdcage coil. The power outgoing from the coil was set in order to obtain a value of B_1^+ in the centre of the coil of 2.5 µT without any metallic object inside the phantom. Finally, the SAR values are normalized to obtain a SAR value of $2\,\mathrm{W\,kg^{-1}}$ averaged over the whole phantom.

Figure S3 regards the results obtained for the clockwise rotating magnetic field B_1^+ and Fig. S4 those obtained for the SAR. The agreement between the two curves is found to be satisfactory for both cases validating all the results proposed in the main paper and in the present Supplementary Material text.

Clockwise rotating Magnetic Field B_1^+

In this section, some of the results referring to the B_1^+ homogeneity, that are not shown in the main article, are proposed. As in the main article, all the proposed figures refer to the ratio, expressed in decibel, between B_1^+ and its maximum value on the slice on which the result is presented.

The presence of the cylindrical metallic object positioned in the centre of the phantom does not perturb the B_1^+ homogeneity neither in “Plane 1” (Fig. S5(a),(b)) nor in “Plane 2” (Fig. S5(c),(d)). In the same way, also the difference in terms of B_1^+ homogeneity between the uncoated (Fig. S5(a),(c)) and coated (Fig. S5(b),(d)) object remains imperceptible. In all these cases, the standard deviation of the clockwise rotating magnetic field evaluated on the whole slice is analogous to that obtained for the unperturbed situation (i.e. without any object inside the phantom).

The same considerations persist dealing with the metallic disc object plunged into the centre of the phantom (Fig. S6). The B_1^+ standard deviation remains comparable to that of the unperturbed case for both the uncoated and coated disc and for all planes shown in Fig. S6. The B_1^+ (dB_{MAX}) is depicted for the realistic hip prosthesis, placed in the centre of the phantom, in Fig. S7. As can be noticed, the prosthesis does not sensibly influence the B_1^+ homogeneity. In particular, there are no significant variation of the standard deviation between

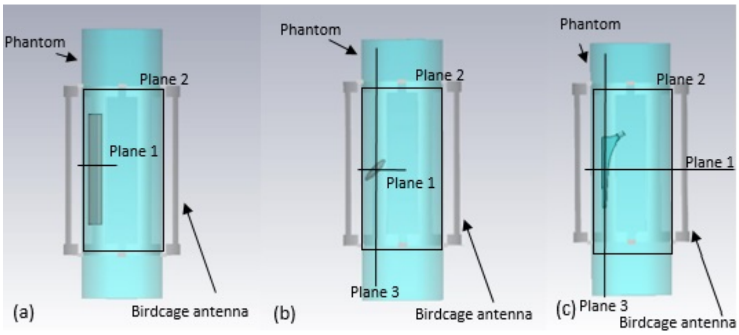

Figure S1: Simulation set-ups relative to the PEC cylinder (a), the PEC disc (b) and the PEC hip prosthesis (c) placed in the lateral position. The planes on which the clockwise rotating magnetic field homogeneity and the SAR were investigated are depicted.

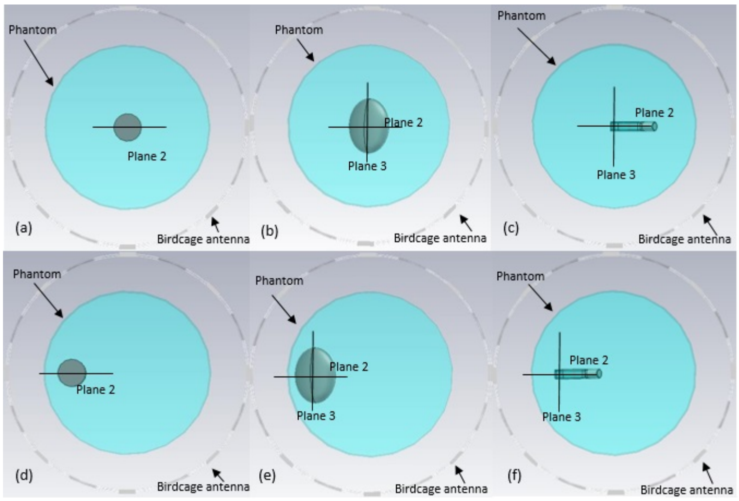

Figure S2: Simulation set-ups relative to the PEC cylinder (a, d), PEC disc (b, e) and PEC hip prosthesis (c, f). The metallic objects were positioned in the phantom centre (a, b, c) and in a lateral position (d, e, f). The planes on which the clockwise rotating magnetic field homogeneity and the SAR were examined are shown.

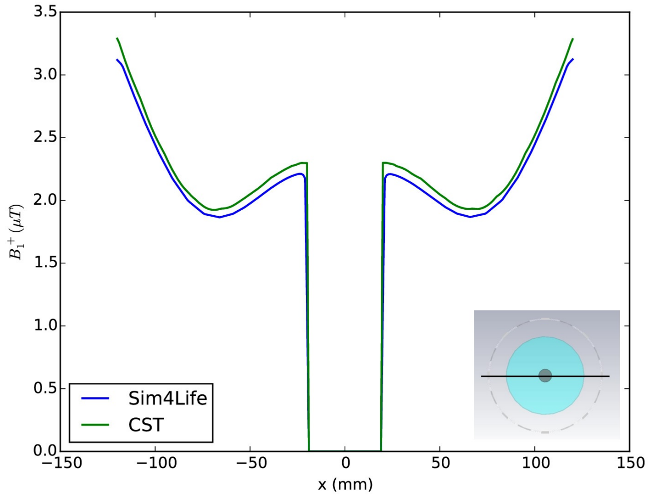

Figure S3: Comparison between the B_1^+ inside the phantom obtained with the two different numerical methods. The B_1^+ is represented along the phantom symmetry transverse line lying on the central transverse plane of the phantom. The investigated line is depicted at the lower right-hand side of the figure.

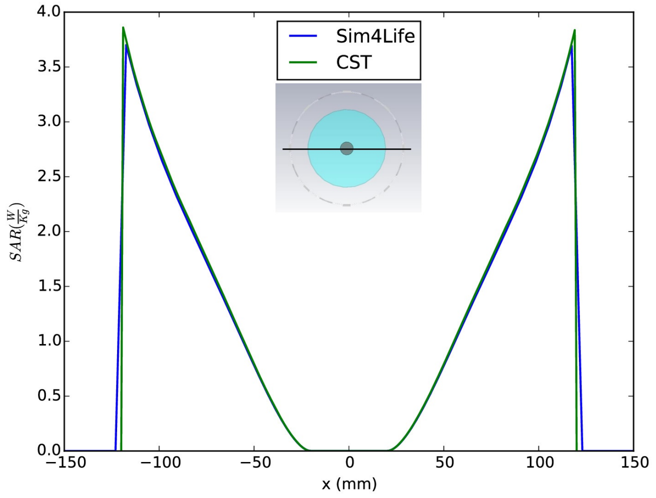

Figure S4: Comparison between the SAR inside the phantom obtained with the two different numerical methods. The SAR is represented along the phantom symmetry transverse line lying on the central transverse plane of the phantom. The investigated line is depicted in the centre of the figure.

the uncoated and coated object for none of the considered slices. Figure S8 shows the coat effects for the disc object plunged into the phantom in a lateral position. The B_1^+ (dB_{MAX}) is depicted on “Plane 3”. The standard deviation of B_1^+ keeps equal to 0.7 µT, both for the uncoated and coated object, if evaluated on the whole slice. Considering a reduced area (x: -60 mm to -60 mm, y: -30 mm to 30 mm), the standard deviation decreases by 33 % passing from 0.9 µT to 0.6 µT.

Specific Absorption Rate (SAR)

In this section, some of the results about the SAR that are not shown in the main paper are proposed and briefly commented. As in the main article, the SAR is always normalized to obtain a volume SAR value of $2\,\mathrm{W\,kg^{-1}}$ averaged over the whole phantom.

Figure S9 depicts the SAR values related to some cases that present critical aspects in the main article. In particular, it is notable that the values of SAR in the slice centre is very low (especially for the unperturbed case) for all the three simulation set-ups shown in Fig. S9. This aspect confirms the assumption that the hight spot values appreciable in the main article (where the SAR is proposed as the ratio between perturbed an unperturbed case expressed in decibel) are due to the low values of SAR and they do not represent a critical situation. The high values of SAR registered near the disc edges (please note the scale of Fig. S9(c),(f)) are due to the tapered shape of the disc and remain limited to a very narrow zone.

Figure S10(a) shows the SAR ratio, expressed in decibel, between the SAR obtained with the coated metallic prosthesis and with the uncoated one for a lateral position. It is appreciable that the points characterized by SAR values higher than those obtained considering the unperturbed case (refer to the main article), present SAR values lower than those characterizing the uncoated cylinder (green zones in Fig. S10). In contrast, the points where the SAR values for the coated cylinder are higher than those for the uncoated case (red zones in Fig. S10) present SAR values lower than those characterizing the unperturbed set-up. It follows that, dealing with the considered set-up, the coat does not bring to SAR values higher than those obtained for both the unperturbed case and the uncoated object case. Figure S10(b) demonstrates that the coated prosthesis does not bring to critical SAR values, on “Plane 2”, when it is plunged into the phantom in a lateral position.

Finally, the ratio between the SAR distribution with the coated metallic hip prosthesis and the one without any object is proposed in Fig. S11. Again, the metallic hip prosthesis seems not to bring sensible SAR variations when the prosthesis is plunged into the phantom centre.

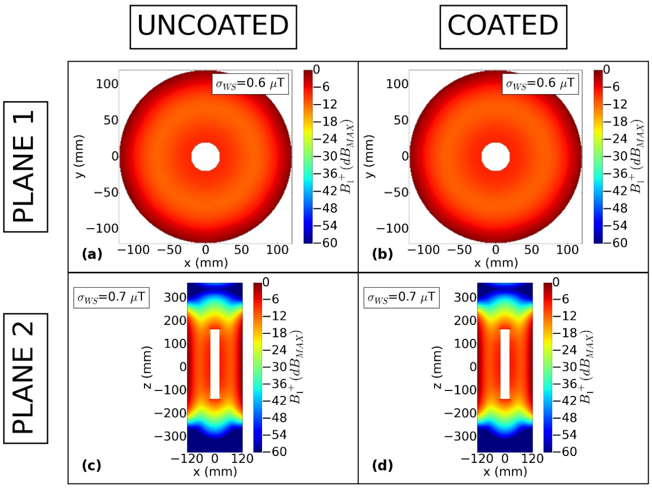

Figure S5: B_1^+ (dB_{MAX}) evaluated for the cylinder plunged into the phantom in the central position. Figures (a) and (b) depict the uncoated and coated object situations, respectively, for the transverse plane that cuts the cylinder in two equal parts along the longitudinal direction. Figures (c) and (d) depict the uncoated and coated object conditions, respectively, for the longitudinal plane that cuts the phantom in two equal parts. σ_{WS} represents the standard deviation computed on the whole slice.

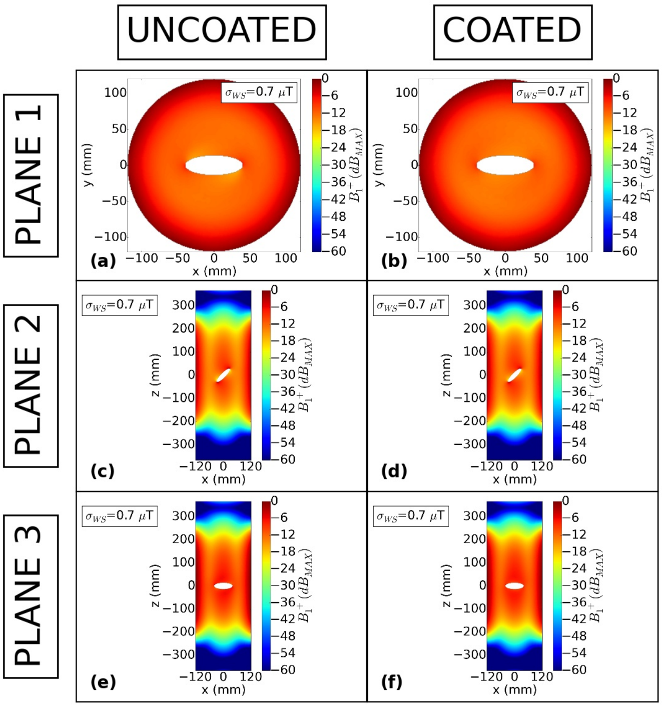

Figure S6: B_1^+ (dB_{MAX}) evaluated for the metallic disc plunged into the phantom in the central position. Figures (a) and (b) depict the uncoated and coated object situations, respectively, for the transverse plane that cuts the phantom in two equal parts along the longitudinal direction. Figures (c),(e) and (d),(f) refer to the uncoated and coated object, respectively, for the longitudinal planes that cut the disc in two equal parts. σ_{WS} represents the standard deviation computed on the whole slice.

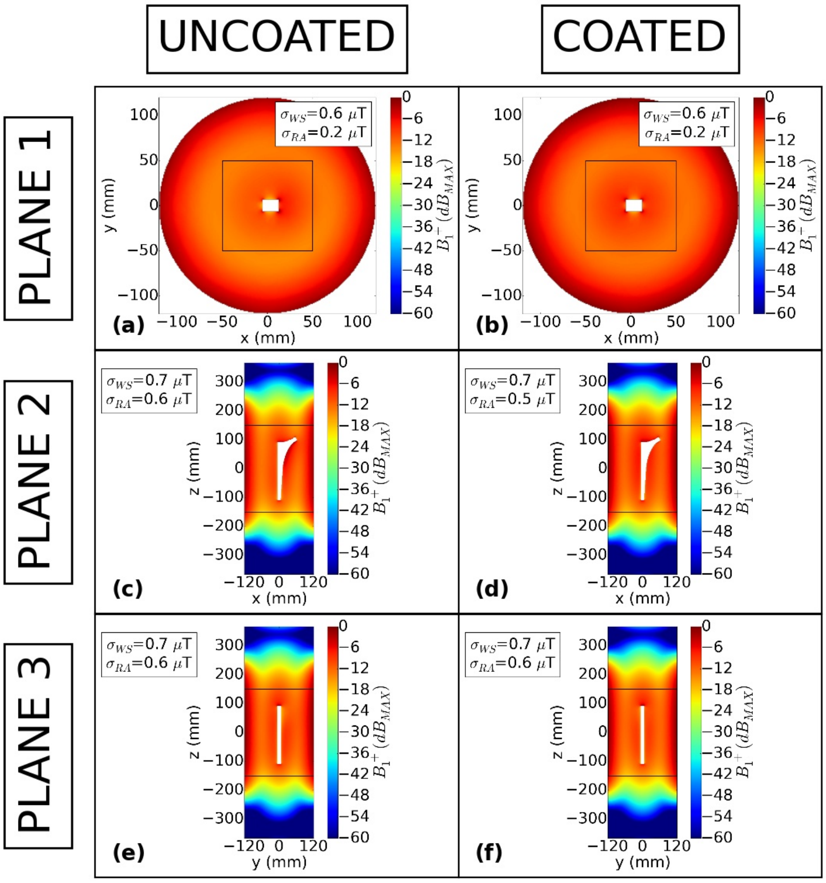

Figure S7: B_1^+ (dB_{MAX}) evaluated for the realistic hip prosthesis model plunged into the phantom in the central position. Figures (a) and (b) depict the uncoated and coated object situation, respectively, for the transverse plane that cuts the phantom in two equal parts along the longitudinal direction. Figures (c) and (d) depict the uncoated and coated object condition, respectively, for the coronal plane that cuts the prosthesis in two equal parts. Figures (e) and (f) depict the uncoated and coated object condition, respectively, for the sagittal plane that cuts the phantom in two equal parts. The rectangular areas represent the reduced areas in which the standard deviation (σ_{RA}) was computed. σ_{WS} represents the standard deviation computed on the whole slice.

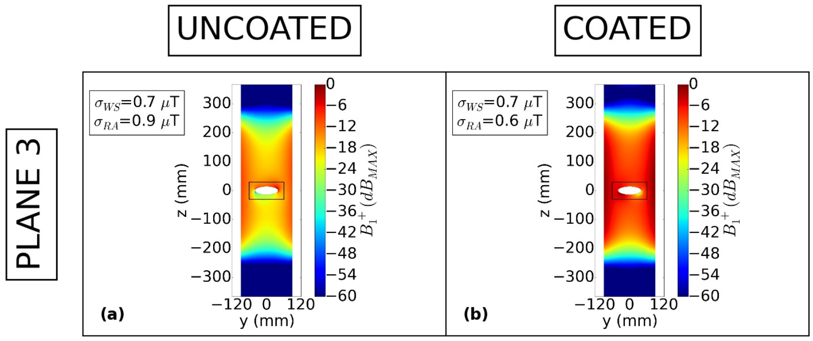

Figure S8: B_1^+ (dB_{MAX}) evaluated for the metallic disc plunged into the phantom in a lateral position. Figures (a) and (b) depict the uncoated and coated object situations, respectively, for a longitudinal plane that cuts the object in two equal parts. The rectangular areas represent the reduced areas in which the standard deviation (σ_{RA}) was computed. σ_{WS} represents the standard deviation computed on the whole slice.

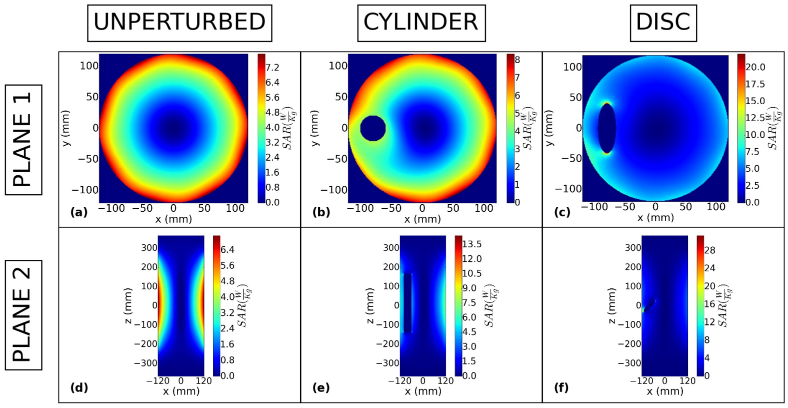

Figure S9: SAR values normalized to obtain a volume SAR value of 2 W kg^{-1} averaged over the whole phantom. The unperturbed situation is reported in (a) for the transverse plane that cuts the phantom in two equal parts along the longitudinal direction (maximum SAR value equal to 7.95 W kg^{-1}). The SAR values for the same plane are depicted in (b) for the lateral coated cylinder (maximum SAR value equal to 8.36 W kg^{-1}) and in (c) for the lateral coated disc (maximum SAR value equal to 22 W kg^{-1}). The same distributions are depicted on the longitudinal planes in (d) (maximum SAR value equal to 7 W kg^{-1}), (e) (maximum SAR value equal to 14 W kg^{-1}) and (f) (maximum SAR value equal to 31 W kg^{-1}) for the unperturbed case, the cylinder and the disc respectively.

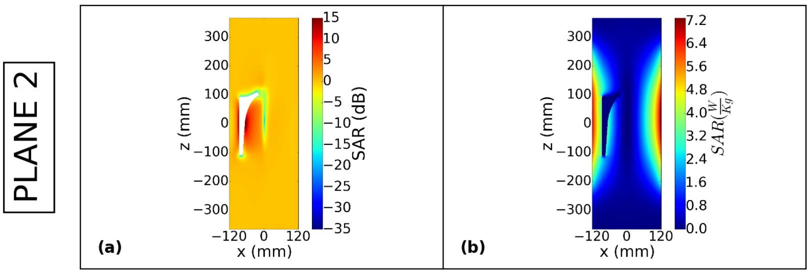

Figure S10: SAR ratio, expressed in dB, between the SAR obtained with the coated metallic prosthesis and with the uncoated one (a) positioned laterally. SAR values normalized to obtain a volume SAR value of $2\,\mathrm{W\,kg^{-1}}$ averaged over the whole phantom in presence of the laterally positioned coated hip prosthesis (b) (maximum SAR value equal to $7.3\,\mathrm{W\,kg^{-1}}$). In both figures, the coronal plane that splits the phantom in two equal parts is considered.

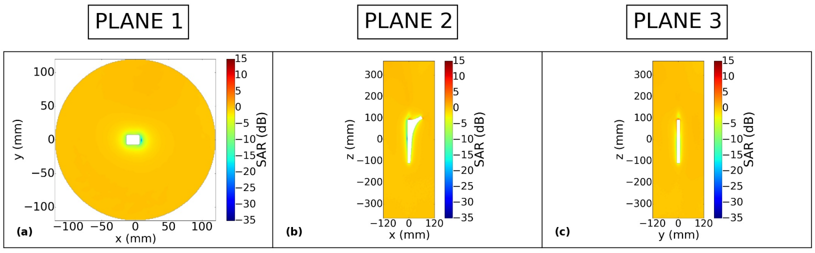

Figure S11: Ratio, expressed in decibel, between the SAR distribution with the coated metallic hip prosthesis inside the phantom and the one without any object. Figures refer to the object plunged into the centre of the phantom. Figure (a) refers to the transverse plane that cuts the phantom in two equal parts along the longitudinal direction. Figures (b) and (d) refer to the coronal and sagittal plane, respectively, that cuts the prosthesis in two equal parts.
